# Thumb Radial Collateral Ligament Reconstruction With Autologous Graft: A Technical Note

**DOI:** 10.7759/cureus.82539

**Published:** 2025-04-18

**Authors:** Konstantinos Mastrantonakis, Georgios Kalinterakis, Eleni Christodoulou, Georgios Psilomanousakis, Christos Yiannakopoulos

**Affiliations:** 1 Orthopaedics and Traumatology, General Hospital of Rethymnon, Rethymno, GRC; 2 School of Physical Education and Sports Science, National and Kapodistrian University of Athens, Athens, GRC; 3 Orthopaedics, IASO Hospital, Athens, GRC

**Keywords:** carpometacarpal joint instability, keyhole ligamentoplasty, ligament injuries, radial collateral ligament, thumb

## Abstract

Several procedures have been described to restore range of motion and stability for chronic radial collateral ligament (RCL) injuries. Anatomical repairs are indicated for acute tears, while for neglected cases, several reconstruction techniques have been described. The purpose of this technical note is to present a detailed, modified surgical technique of treating a chronic RCL tear, using an autologous tendon graft placed in a triangular fashion using drill holes (keyhole technique).

## Introduction

Rupture of the radial collateral ligament (RCL) of the first metacarpophalangeal (MCP) joint of the thumb is a rare injury that needs greater attention. The incidence of RCL injuries in the literature ranges from 10 to 42% of all collateral ligament injuries [1]. Despite its rarity, it constitutes damage to an important structure, which can cause instability of the thumb MCP joint. Neglected cases can lead to instability, progressive osteoarthritic changes, reduced grip and pinch strength, and hand disability [1-3]. In laborers, neglected injuries lead to time off work, while in athletes, it can result in prolonged time for return to play [4]. Clinical examination and proper history of the injury are critical, and these will determine the treatment option to be followed [5].

Surgical treatment is recommended according to recent literature data, particularly in the setting of rotational deformity with volar subluxation after casting. For articular avulsion fractures with involvement of greater than 30% of the articular surface, open fixation is recommended. Treatment options include open repair, RCL reefing, and reconstruction with free tendon graft [6]. The palmaris longus is often a standard option and, if absent, common alternative autogenous sources include advancement of the abductor pollicis brevis [7], or a strip of flexor carpi radialis (FCR) or, scarcely, an extensor tendon [8,9]. In this technical report, we propose a ligament reconstruction technique with free autograft based on the keyhole tenodesis of the long head of the biceps [10].

## Technical report

**Figure 1:**
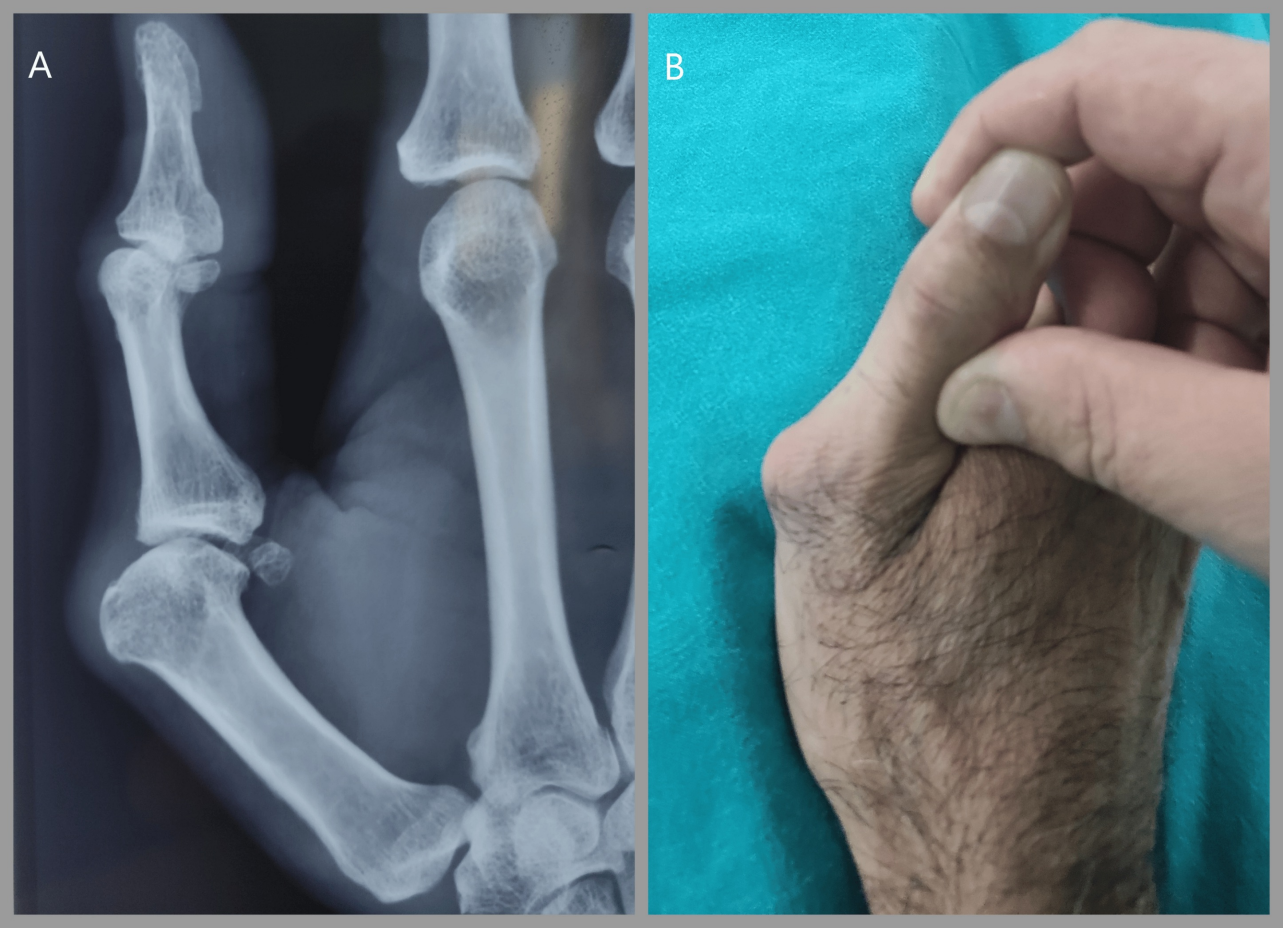
Preoperative images A: Plain lateral radiograph showing volar displacement of the proximal phalanx in relation to the metacarpal. B. A dorsoradial prominence was noted at the metacarpophalangeal joint

A 40-year-old male presented with pain over the proximal right thumb for several months, aggravated by movements when grasping objects and during everyday hand activities. A dorsomedial prominence was noted (Figure 1B). He had suffered a closed thumb injury a year ago and underwent splint treatment for four weeks at that time. There was significant laxity in the joint resistance test in flexion and extension compared to the healthy MCP articulation. Radiographic evaluation included standard anteroposterior, lateral, and oblique views. Plain lateral radiographs showed volar displacement of the proximal phalanx in relation to the metacarpal (Figure 1A). No damage to the articular cartilage was seen on the preoperative X-rays.

Surgical technique

**Figure 2:**
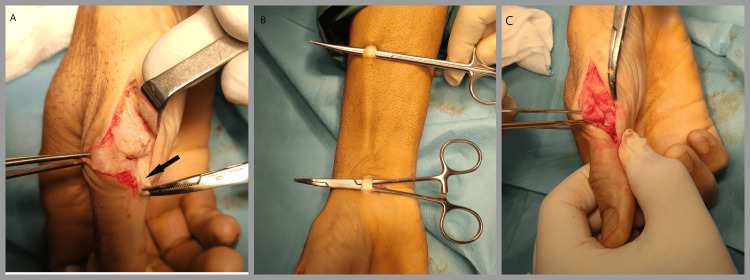
Perioperative images - 1 A. The abductor aponeurosis was retracted volarly, and the joint capsule was incised. B. From two 1 cm incisions proximal and distal to the forearm, a 15 cm strip from the FCR tendon graft was harvested. C. The two tunnels were made at the base of the phalanx (about 5 mm from the articular surface) when viewed from the side of the right thumb, so they create a tunnel with a sufficient distance between them so that no bone is detached when the graft slides FCR: flexor carpi radialis

With the patient in the supine position, the upper extremity was supported on an auxiliary table. A tourniquet was used at a pressure 100 mmHg above the systolic pressure of the patient. Α radial lateral incision over the MCP joint, between the palmar skin folds and the dorsal surface of the thumb, was used. The edges of the skin were raised, and care was taken to identify and protect the superficial branches of the radial nerve. The dissection was between the extensor hood and extensor tendons, specifically the extensor pollicis longus (EPL) and extensor pollicis brevis (EPB). The head of the first metacarpal was identified along with the base of the proximal phalanx, which were covered with scar tissue. The abductor aponeurosis was retracted volarly, and the joint capsule was incised (Figure 2A). The area where the RCL should have been was replaced by fibrous tissue, which was removed to create the tunnels. In addition, the dorsolateral osteophyte was removed as well.

Graft Harvest

On the volar side of the wrist, through two 1 cm separate incisions, a 15 cm strip of FCR tendon was harvested for the reconstruction (Figure 2B). Fibrotic tissue from the base of the head of the metacarpal and the base of the proximal phalanx was removed. A 2.7 mm diameter drill was used to create three tunnels. Two sequential tunnels were created at the base of the phalanx (about 5 mm distal to the articular surface) perpendicular to the longitudinal axis of the bone and with a sufficient distance between them so that no bone is detached when the graft slides (Figure 2C). The third tunnel was created at the head of the metacarpal bone (5 mm proximal to the articular surface) with direction radial to ulnar and perpendicular to the longitudinal axis of the metacarpal bone.

**Figure 3:**
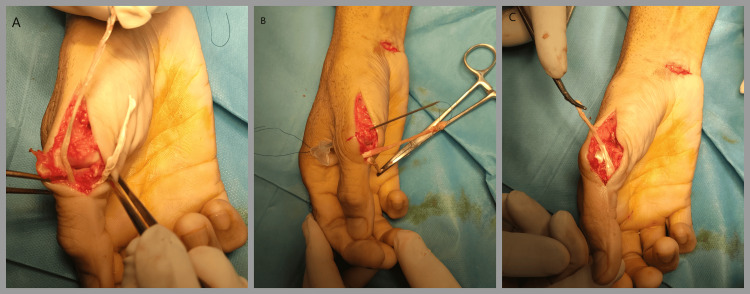
Perioperative images - 2 A. The sutures were connected to one end of the graft and passed sequentially, first through the phalanx tunnel. B. A small counter-incision was performed in the ulnar side of the metacarpal, and the suture ends were passed through the ulnar cortex of the metacarpal through an epidural catheter tube. C. Then, the radial end of the tendon graft was pulled with the metacarpophalangeal joint in an abducted position, flexed at 45 degrees

**Figure 4:**
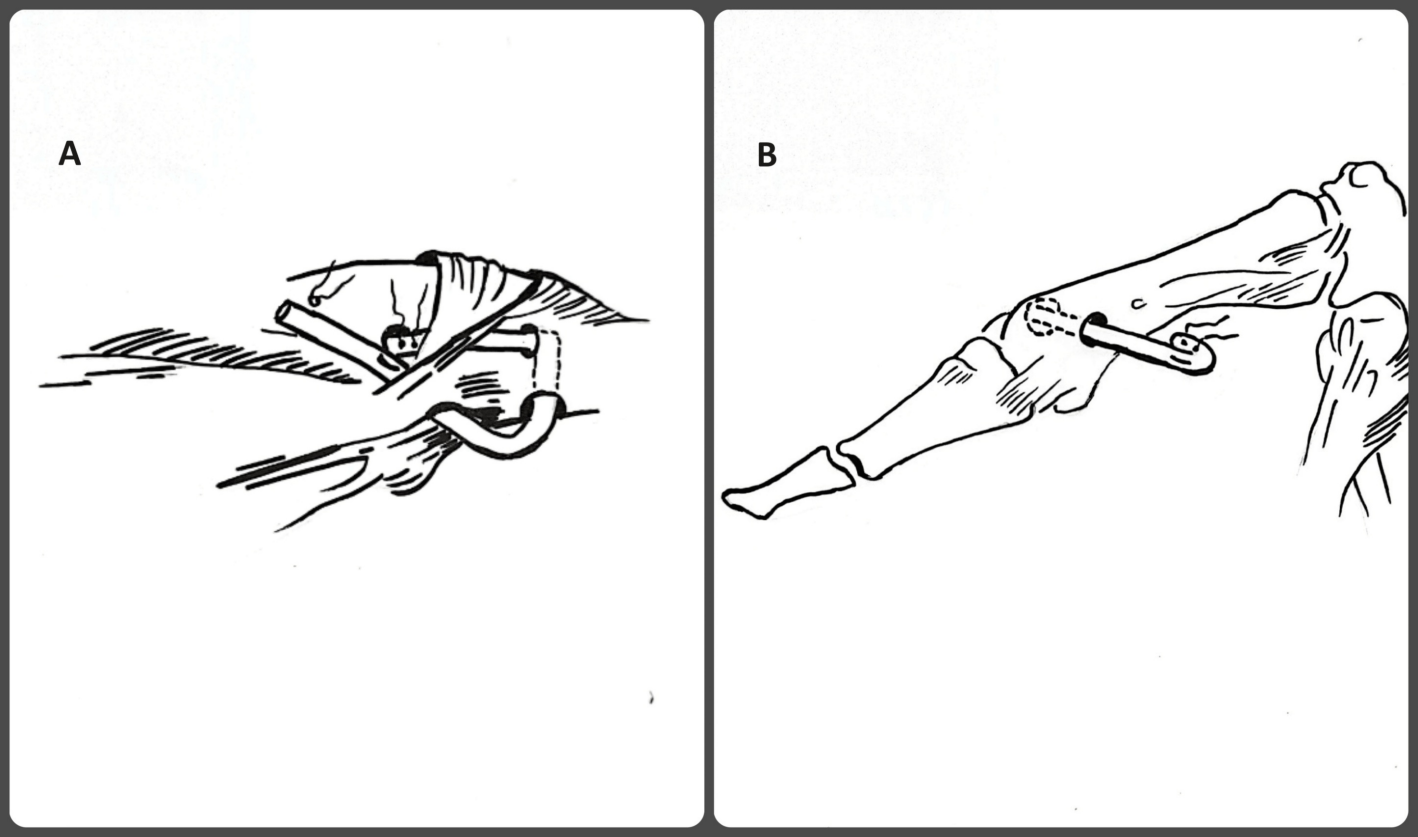
A. The graft limbs at the radial side were docked together with non-absorbable suture and tensioned to the metacarpophalangeal bone with a mini–suture anchor adjacent to the radial hole. The tendon graft was then secured with periosteal sutures in the graft tails at the ulnar cortex. B. The ulnar end of the tendon was folded back and sutured with non-absorbable sutures creating a plug

A suture was passed through the tunnel of the proximal phalanx, and a second one through the metacarpal with the help of an epidural catheter tube after first incising the skin from the ulnar side of the metacarpal bone. The sutures were connected to one end at the graft and passed sequentially, first through the phalanx tunnel and then through the metacarpal tunnel, creating an inverted triangle (Figure 3B). The ulnar end of the tendon was folded back and sutured with non-absorbable sutures, creating a plug (Figure 4B). Then, the radial end of the tendon graft was pulled with the MCP joint in an abducted position, flexed at 45 degrees (Figure 3C). The graft limbs at the radial side were docked together with a non-absorbable suture and tensioned to the MC bone with a mini-suture anchor adjacent to the radial tunnel (Figure 4A).

The capsule was closed with absorbable sutures over the graft, and the skin was closed with a subcuticular absorbable suture. The thumb was immobilized in a splint for five weeks. The patient had regular weekly follow-up appointments and wound dressing changes. At the end of the fifth week post-op, he was referred to a physiotherapist for rehabilitation.

## Discussion

RCL injuries are rare entities, and their inappropriate treatment or misdiagnosis can lead to joint instability and disability. In cases where the injury is diagnosed, stabilization of the thumb MCP joint can lead to adequate healing of the ligament. Partial tears and tears that do not cause subluxation and rotational deformity can heal with immobilization in a forearm splint for four to six weeks. The main difference between the radial and ulnar sides of the MCP joint is the involvement of the abductor's tendon insertion. In ulnar collateral ligament tears, there is a possibility that the abductor's aponeurosis may be involved between the tears (Stener lesion), thereby making healing difficult. In RCL tears, there is usually no interfering tendon similar to the Stener lesion, although it has been reported [10].

With the absence of a Stener’s equivalent radially, the role of immediate repair is not well defined. Some authors recommend casting of acute RCL tears, and others recommend surgical repair. In acute complete tears, there is no fixed point of resistance on the ulnar stress test. In chronic tears, there is subluxation of the joint, with dorsolateral prominence of the head of the metacarpal bone, weakness, and inability to grasp. According to the data in the literature, surgery is the recommended treatment, particularly if rotational and with volar subluxation after casting. For articular avulsion fractures with involvement of greater than 30% of the articular surface, open fixation is recommended. There is a lack of evidence regarding an absolute indication for surgery in complete ruptures. However, in clinical practice, surgery is always recommended when there is no stable point of resistance and instability is observed. Acute tears should be repaired immediately, especially when there is volar subluxation of the proximal phalanx. Patients with chronic tears and instability should always be offered reconstruction with a graft.

There are several techniques for ligament reconstruction, more often using the ipsilateral palmaris longus as a graft. Edelstein et al. have described a ligament reconstruction technique using an autologous graft. The tendon was placed across the radial side of the MCP joint in a triangular fashion using small drill holes in the base of the thumb proximal phalanx [1]. The graft was tensioned and secured with a suture anchor or by tying the graft tails together on the ulnar side through a tunnel in the metacarpal neck [1,3]. Other authors have described reconstruction techniques with the use of a slip of the abductor pollicis brevis. In a cadaver study by Schmidt et al., a slip of the abductor pollicis brevis tendon was dissected and reattached proximally [7]. A similar technique has been described by Horch et al., in which a slip of abductor pollicis brevis was redirected to reconstruct the radial-sided restraint of an RCL-deficient thumb MCP joint. They reported high patient satisfaction with good joint motion and improved stability at a mean follow-up of 39 months in a series of nine patients with a combination of acute and chronic injuries [11].

Τhe keyhole technique was first described in 1975 by Froimson and is used in tenodesis of the long head of the biceps [12]. Its stability is attributed to the fact that the tendon is reversed and a plug is created, which works like an Endobutton. Later, the technique was modified by other authors with the use of arthroscopy, with impressive results in terms of stability and success rate [13]. In the above-described technique, we used the folding of a free tendon graft to create a plug exactly like the keyhole technique. In this way, we created a fixed point that replaced an anchor or an Endobutton.

## Conclusions

Keyhole ligament reconstruction technique is a safe, easy, reliable, and cost-effective surgical treatment. We propose it for subacute or chronic instability of the thumb MCP joint and in cases where primary ligament repair is not feasible. The use of the mini-suture anchor is optional and could be omitted to lower the treatment cost. We believe that this ligament reconstruction technique, based on keyhole tenodesis, could be used for ligament reconstruction in other joints as well.
